# Cue combination for depth perception in macular degeneration: Motion parallax augments disparity

**DOI:** 10.1167/jov.26.1.11

**Published:** 2026-01-22

**Authors:** Jade Guénot, Preeti Verghese

**Affiliations:** 1 Smith-Kettlewell Eye Research Institute, San Francisco, CA, USA

**Keywords:** central field loss, macular degeneration, depth perception, disparity, motion parallax, cue integration

## Abstract

In macular degeneration (MD), depth perception from binocular disparity is impacted in regions with vision loss in either eye, but monocular cues like motion parallax remain available. This study investigates whether combining motion parallax with disparity improves depth perception and compensates for the loss of depth due to central field loss (CFL). Eleven MD participants and 19 controls viewed a horizontal sine-wave corrugation in depth, defined by disparity and/or motion parallax, judging which half-cycle appeared farther away in depth. We measured thresholds for each cue alone and for the two cues combined. In MD participants, cue integration benefits depended on scotoma characteristics. Disparity performance correlated strongly with the size of the stereoblind zone, while motion parallax thresholds showed no significant relation, suggesting preservation despite CFL. MD participants with extensive stereoblind zones showed elevated thresholds for both single cues compared to controls but demonstrated optimal integration when disparity was added to motion parallax. Those with small stereoblind zones achieved control-like thresholds and exhibited optimal or better than predicted integration. However, asymmetric patterns emerged with suboptimal performance when motion parallax was added to threshold disparity. Controls with simulated scotomas maintained stable integration, contrasting with variable patterns in MD. Our results show that individuals with CFL retain significant capacity for depth cue integration, contingent upon residual binocular disparity. Thus, motion parallax emerges as a valuable compensatory cue to improve depth perception in individuals with MD.

## Introduction

The perception of depth is essential to navigate in our three-dimensional world and judge the distance of objects accurately. Binocular disparity is often considered the most robust and reliable visual cue for depth perception. It refers to the slightly different images of the world projected onto each retina, which allow the perception of depth from disparity, known as stereopsis (Qian, 1997; Wheatstone, 1997).

In normally sighted viewers, fine stereopsis is achieved in the fovea, and its precision decreases with eccentricity (Fendick & Westheimer, 1983; Siderov & Harwerth, 1995). In individuals with central field loss due to macular degeneration (MD), depth perception is compromised (Cao & Markowitz, 2014; Demir, Kayhan, Sevincli, & Sonmez, 2023; Verghese, 2023; Verghese & Ghahghaei, 2020 Verghese, Ghahghaei, & Lively, 2022). The loss of stereopsis is not limited to the fovea but extends to all the parts of the visual field that correspond to the scotoma in either eye, even when only one eye is affected (Verghese et al., 2022). In areas peripheral to the scotoma, coarse stereopsis remains intact (Verghese et al., 2022) and can still be useful for everyday tasks that involve depth perception, such as walking (Bonnen et al., 2021; Buckley, Panesar, MacLellan, Pacey, & Barrett, 2010; Hayhoe, Gillam, Chajka, & Vecellio, 2009) or eye–hand coordination (Cao & Markowitz, 2014; Melmoth, Finlay, Morgan, & Grant, 2009; Verghese, Tyson, Ghahghaei, & Fletcher, 2016).

However, the visual system does not rely exclusively on binocular disparity for relative distance and depth judgments. A multitude of monocular cues, such as linear perspective (Saunders & Backus, 2006), texture gradients (Gibson, 1950; Srikakulapu, Kumar, Gupta, & Venkatesh, 2015), blur (Watt, Akeley, Ernst, & Banks, 2005), relative size (Fineman, 1971), lighting and shading (Kim & Anstis, 2016; Lovell, Bloj, & Harris, 2012), and motion parallax (Ono, Rivest, & Ono, 1986; Rogers & Graham, 1979; Rogers & Graham, 1982), also contribute to our perception of depth. These binocular and monocular cues are combined and weighted to maximize the precision and accuracy of depth estimates, especially when dealing with complex scenes where individual depth cues may be ambiguous (Landy, Maloney, Johnston, & Young, 1995).

In individuals with MD, using monocular depth cues could provide a useful strategy for extracting more precise depth information to compensate for impaired binocular disparity processing. Indeed, many individuals with MD present with asymmetric visual deficits between the two eyes in terms of visual acuity, contrast sensitivity, and size and shape of their scotomas, and they may rely on their better eye to extract monocular depth information. This may be particularly relevant for individuals who have lost central stereopsis due to a monocular scotoma, when the better eye is intact.

Among the various monocular cues, motion parallax seems particularly promising. This unambiguous cue refers to the relative motion between objects at different depths (Howard & Rogers, 1995) and can be created either by observer head translation relative to the scene (Steinbach, Ono, & Wolf, 1991; Ujike & Ono, 2001), the scene translation relative to a stationary observer (Nawrot & Joyce, 2006), and/or during pursuit eye movement (Naji & Freeman, 2004; Nawrot, 2003; Nawrot & Joyce, 2006). Critically, unlike other monocular cues like lighting and shading, motion parallax is not based on sensitivity to contrast, which is impaired in individuals with MD (see, e.g., Hogg & Chakravarthy, 2006), but on the perception of motion, which is preserved in this clinical population (Guénot et al., 2022; Shanidze & Verghese, 2019). Moreover, Bradshaw and Rogers (1996) showed that for normally sighted observers, the presence of both binocular disparity and motion parallax reduced the threshold to detect the three-dimensional structure of corrugated surfaces by 48% on average, relative to the most sensitive single-cue condition (typically disparity alone). This subthreshold summation indicates a facilitation in normally sighted viewers, but to date, no study has explored whether it is also present in individuals with central field loss.

Therefore, this study aims to determine whether combining disparity and motion parallax can improve depth perception and compensate for the loss of fine binocular depth cues when vision loss occurs in the central retina in one or both eyes, as well as whether individuals with MD are still able to combine these two cues optimally. We used stimuli consisting of random dots made up of a horizontally oriented sine-wave corrugation in depth, defined by disparity and/or motion parallax to measure thresholds in participants with MD and in controls with no scotoma, as well as with simulated scotomas of 4°, 6°, and 8° diameter.

## Methods

### Participants

Eleven participants with macular degeneration (aged 62–92 years, 7 women) and 19 control participants (aged 25–82 years, 8 women) were included in this study. Control participants had normal vision, a visual acuity better than 0.1 logMAR (logarithm of the minimum angle of resolution) in both eyes, and stereoacuity better than 200 arcsec. Their stereoacuity was measured using the Asteroid stereotest (Vancleef et al., 2019). While most young adults have stereoacuity better than 100 arcsec (Coutant & Westheimer, 1993), choosing a 200-arcsec threshold ensured the inclusion of sufficient older participants, given that stereoacuity declines with age (Schneck, Haegerstrom-Portnoy, Lott, & Brabyn, 2000). Ten of the participants with MD had age-related macular degeneration, and one had Stargardt's disease. For participants with MD, coarse stereoacuity was measured with the Random Dot Stereo Butterfly Test. Their preferred retinal locus (PRL) eccentricity and stereo scotoma sizes were estimated using the Macular Integrity Assessment (MAIA; CenterVue, Padova, Italy), as described in the next sections. Table 1 reports the individual clinical characteristics of all participants with MD.

**Table 1.** Individual clinical characteristics of the participants with MD included in the study. *Note*: AMD = age-related macular degeneration; logMAR = logarithm of the minimum angle of resolution; OD = right eye; OS = left eye; PRL eccentricity of DE: preferred retinal locus of dominant eye.

					Visual acuity (logMAR)					
Subject	Age, y	Sex	Diagnosis	Stereoacuity (arcsec)	OS	OD	Dominant eye	PRL eccentricity of DE	Stereo scotoma size (max x and y)	Binocular scotoma size (max x and y)
MD1	83	F	AMD	200	0.74	0.36	OD	1.36°	11.41° × 13.81°	9.62° × 6.91°
MD2	83	F	AMD	>3,000	0.92	0.86	OD	1.63°	24.74° × 18.06°	11.30° × 14.52°
MD3	62	M	Stargardt	1,150	1.1	1.2	OD	5.24°	21.46° × 11.77°	15.18° × 9.67°
MD4	81	F	AMD	>3,000	0.86	0.96	OS	6.49°	21.28° × 20.11°	15.05° × 15.06°
MD5	83	F	AMD	>3,000	0.8	0.46	OD	1.17°	23.56° × 18.07 °	5.44° × 5.66°
MD6	75	F	AMD	100	0.82	0.14	OD	1.67°	Relative scotoma	No binocular scotoma
MD7	92	M	AMD	100	0.1	0.6	OS	3.25°	3.42° × 2.87°	No binocular scotoma
MD8	72	F	AMD	1,200	0.32	0.3	OD	1.51°	7.29° × 5.35°	No binocular scotoma
MD9	71	F	AMD	160	0.36	0.34	OD	0.39°	Relative scotoma	No binocular scotoma
MD10	81	M	AMD	>3,000	1.08	0.4	OD	1.67°	4.27° × 6.34°	No binocular scotoma
MD11	87	M	AMD	>3,000	1.14	>2.0	OS	12.43°	>20° × >20°	7.3° × 4.38°

All experimental procedures were approved by the Institutional Review Board of the Smith-Kettlewell Eye Research Institute and followed the ethical standards of the Declaration of Helsinki. All participants gave informed written consent and received monetary compensation for their participation.

### Measurement of the stereoblind zone

For each individual with MD, the goal was to map the region of inferred stereo loss, which corresponds to the union of the two monocular scotomas (Verghese et al., 2022), and to measure the extent of this stereoblind zone, which we refer to as the *stereo scotoma*. First, we mapped the monocular scotoma in each eye and estimated the position of the fovea and the PRL relative to the fovea. Second, we superimposed the monocular scotoma maps aligned on the fovea and measured the size of the union of the two monocular scotomas. More details are provided in the following sections.

#### Monocular scotoma

Scotoma maps for each eye were obtained by first performing monocular microperimetry in each eye using MAIA with a field size of 36.5°. We used a custom perimetric grid to manually place the test loci on the retina to map the scotoma more finely. Before stimulus presentation, the MAIA system automatically recorded 10 seconds of fixation to estimate the participant's initial PRL and fixation stability. Scotoma mapping was then performed with the default 4-2 staircase strategy (maximum illumination: 318.47 cd/m², attenuation range of 1 dB from 0 to 36 dB, presentation time: 200 ms). Individuals were instructed to fixate a small red circular fixation target while a 25 Hz eye tracker controlled for fixation losses, detected and corrected ocular movements, and registered fixation patterns. The final position of the PRL was estimated by calculating the center of the fixation points recorded during the exam. Retinal threshold sensitivity was estimated for each test locus, with a threshold of 0 dB when the maximum intensity was not seen, corresponding to an absolute scotoma. Testing each eye took 5 to 10 minutes. To define the extent of the monocular scotoma, we then used MATLAB (R2020b; MathWorks, Natick, MA, USA) to select all tested loci with a threshold less than 8 dB and draw the contour of the scotoma. We used the 8-dB criterion as in Verghese et al. (2022), based on the relation between this criterion and Weber contrast in the Optos SLO. In the MAIA CenterVue SLO used here, the background luminance is much lower, and an 8-dB threshold corresponds to a Weber contrast of about 100 (Arango, Morse, & Seiple, 2018). Weber contrast thresholds for controls in the MAIA are around 0.33, making the 8-dB criterion roughly 300 times higher than the normal threshold. Therefore, the 8-dB cutoff provides a conservative criterion to define a monocular scotoma in the context of stereopsis, especially given that binocular disparity is very sensitive to interocular differences in contrast (Legge & Gu, 1989).

#### Fovea and PRL position

The MAIA does not allow for the estimation of both the eccentric PRL and the fovea. To obtain the location of the foveal pit, we used the radial scan function of the Optos optical coherence tomograph (Optos OCT/SLO; Optos, Marlborough, MA, USA), with a field size of 29.7°. (Note that we did not use the Optos for the perimetry due to a misalignment in the SLO.) If the progression of the disease prevented us from locating the fovea, we used the average distance from the optic nerve (15.5° horizontally and –1.5° vertically; Rohrschneider, 2004). The position of the fovea for each eye was then reported on the MAIA images using retinal landmarks, and the coordinates of the PRL were calculated relative to the fovea.

#### Binocular and stereo scotoma

**Figure 1.**
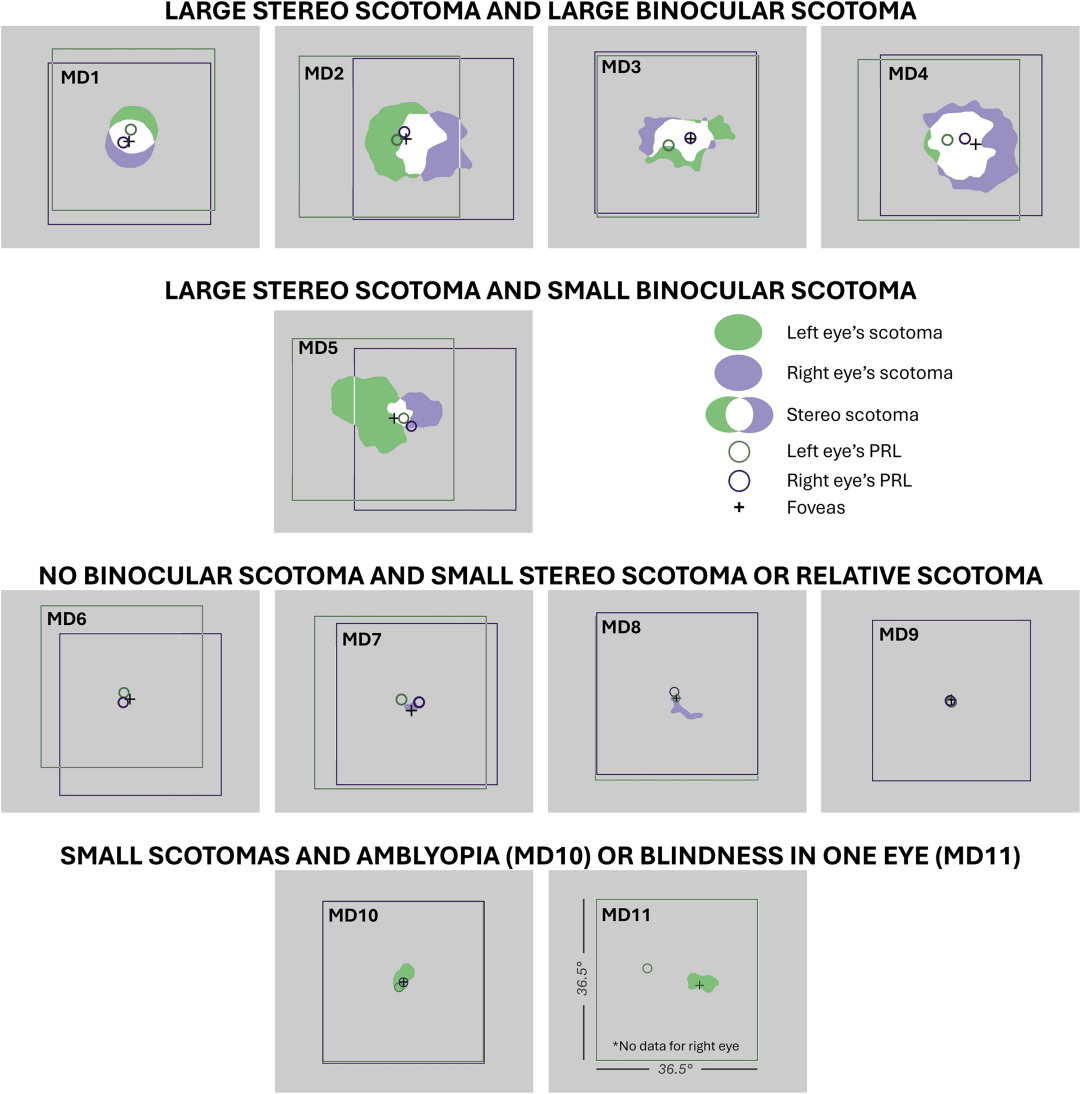
Stereo scotomas (i.e., stereoblind zone) of participants with macular degeneration included in the study. Each square corresponds to an area of 36.5° × 36.5°. For each MD individual, green and purple regions respectively show the left and right eyes’ scotomas. White areas correspond to binocular scotomas (the overlap of the two monocular scotomas). The union of green, white, and purple corresponds to the stereo scotomas. Green and purple circles represent the PRLs for the left and right eye, and the black cross indicates the position of the foveas. Participants MD1 to MD4 have large binocular and stereo scotomas, MD5 has a large stereo scotoma but a small binocular scotoma, MD6 to MD9 have either a small monocular and stereo scotoma or a relative scotoma, and MD10 and MD11 have compromised binocular function due to amblyopia with strabismus (MD10) or blindness in one eye (MD11).

Once we had the monocular scotoma and the fovea and PRL positions, the binocular scotoma and the region of inferred stereo loss were calculated following the methods of Verghese et al. (2022; see also Verghese & Ghahghaei, 2020). It has been shown that individuals with MD tend to align the direction of their binocular gaze with the PRL of their dominant eye (Kabanarou et al., 2006; Tarita-Nistor, Brent, Steinbach, & González, 2012). Thus, we superimposed the retinal maps, aligned on the foveae. The presumptive stereo scotoma (i.e., the stereoblind zone where there is vision loss in either eye) corresponds to the union of the two monocular scotomas (shown in green for the left eye and violet for the right eye, see Figure 1). The binocular scotoma, not to be confused with the stereo scotoma, is the intersection (region of the overlap) between the two scotomas (shown in white, Figure 1) where there is vision loss in both eyes. The values reported in Table 1 for the size of the stereo scotoma represent the size of the region of the visual field with stereo loss.

### Materials

#### Apparatus

**Figure 2.**
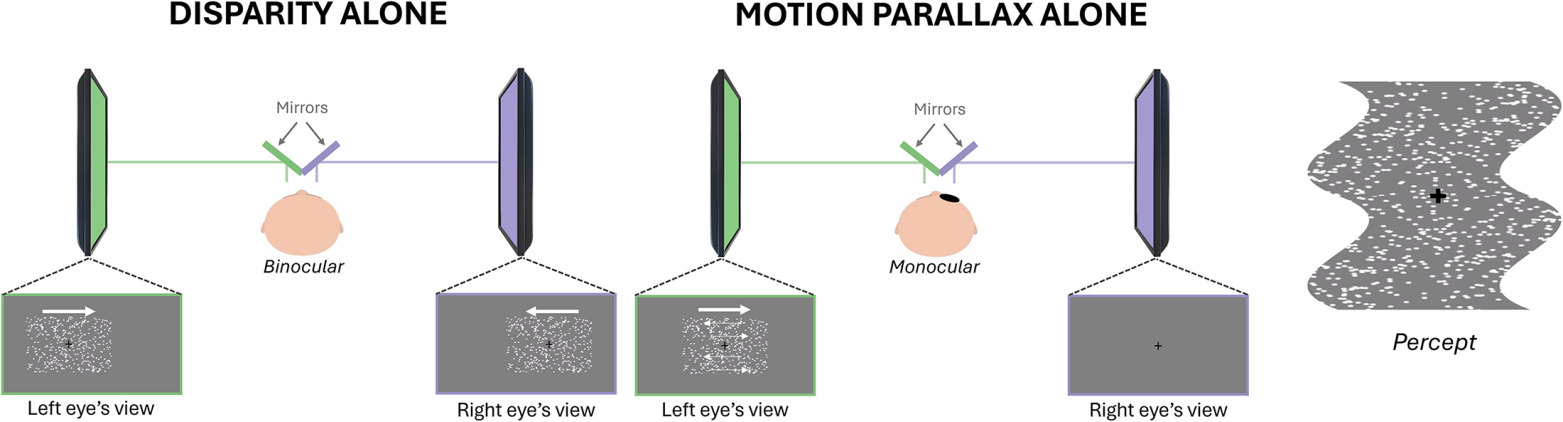
Experimental setup. The left part of the figure shows the “disparity alone” condition. Stimuli are presented on a pair of monitors arranged as a mirror stereoscope, allowing us to project slightly different images to each eye. The central part of the figure illustrates the “motion parallax–alone” condition. The stimuli are presented to the eye with the better visual acuity or to the dominant eye, while the other eye is covered. The right part of the figure illustrates the perception of the corrugated surface in depth during the task.

Stimuli were presented on a pair of monitors arranged as a mirror stereoscope (Figure 2; viewing distance: 68.5 cm, resolution: 1,920 × 1,080 pixels, refresh rate: 60 Hz). Participants had their head stabilized with a head-and-chin rest and responded via a keyboard. During the experiment, the eye with the better visual acuity (or the dominant eye when visual acuity was similar in both eyes, determined by the Miles test; Miles, 1929) was tracked at a 1,000 Hz sampling rate with the table-mount configuration of the eye tracker (Eyelink 1000; SR Research, Ontario, Canada), positioned behind cold mirrors.

#### Stimuli

The stimuli were generated in MATLAB (R2020b) using the Psychophysics toolbox. They depicted two vertical cycles of a corrugated surface, undulating away and toward the observer (see right side of Figure 2). Within a 10.57° × 10.57° square stimulus window, 6,000 nonoverlapping white random dots (0.1° diameter, 56.42 dots/deg² density, 33.2% Michelson contrast; unlimited lifetime) were displayed on a gray background along with a central 0.22° black fixation cross to foster binocular fusion. The dots were used to generate a sine-wave profile in depth with a frequency of 0.2 cpd, defined by disparity and/or motion parallax. Note that 0.2 cpd is close to the optimal spatial frequency for detecting both binocular disparity and motion parallax (Rogers & Graham, 1982).

During the experiment, the stimulus window translated horizontally across the monitors at 3°/s (including when the disparity cue was presented alone). Observers were asked to track the fixation cross, positioned at the center of the aperture, with smooth pursuit eye movements. Accurate pursuit ensured that depth from motion parallax was only generated by relative retinal motion of the dots (Nawrot, 2003; Nawrot & Joyce, 2006), as head-relative motion cues were minimized by the use of a chin and forehead rest. Each trial lasted 3.62 seconds and began with the window centered horizontally and vertically on the screens, translating randomly to the left or right over 5.43° (6.5 cm) before reversing direction and returning to the start position. The sign and magnitude of the depth step were kept constant within a trial (e.g., the part just above the fixation cross was perceived away from the observer during the whole trial).

At the beginning of each trial, random coordinates were assigned to each dot inside the aperture. For disparity-defined depth, the dots presented on each screen were displaced horizontally in opposite directions for right- and left-eye stimuli. A vertically oriented sinusoidal function was used to define the horizontal displacement of each individual dot. Dots representing peaks and valleys of the corrugations had the maximum displacement, with the perceived depth magnitude increasing with the displacement of the dots. The phase of the sine-wave corrugation was randomly assigned on each trial, with two possibilities: either peaks or valleys below the fixation point. The fixation cross and the dots placed on the horizontal line through fixation always had zero disparity.

For motion parallax, the sinusoidal function was used to define the speed of the individual dots. Dots representing peaks and valleys of the corrugations had the highest speed, and the other dots’ velocities were derived from the function. Inside the stimulus window, individual dots translated horizontally in the opposite direction for peaks and valleys. Dots in the half-cycle of the stimulus moving in the opposite direction of pursuit were supposed to be perceived as farther away (Nawrot & Joyce, 2006). As with disparity stimuli, the fixation cross and horizontally aligned dots had zero speed relative to the aperture, and greater peak speed increased perceived depth.

#### Procedure

Participants viewed stimuli monocularly for the motion parallax condition and binocularly for the other conditions. In the monocular condition, the eye with the better visual acuity or the dominant eye when the visual acuity was similar in both eyes was tested while the other eye was covered with a patch. This choice was motivated by pilot observations: When the motion parallax stimulus was viewed binocularly, some participants experienced unstable depth percepts (e.g., a region of the stimulus appeared farther away, then closer to the observer during the trial, even though the depth of that region was held constant). This instability was likely due to a cue conflict between motion parallax information signaling depth and the absence of binocular disparity signaling a flat surface. For all participants, monocular viewing eliminated this instability. For a subset of control participants who did not experience instability under binocular viewing, no differences were found between monocular and binocular performance for the motion parallax–alone condition (*n* = 7).

Participants performed a two-alternative forced-choice depth judgment task and had to determine whether the half-cycle of the stimulus immediately above or below the fixation cross appeared farther in depth. They had unlimited time to respond. Thresholds (75% correct detection) were estimated using the psi-marginal adaptive method (Prins, 2013), which adaptively updates the stimulus level after each trial to maximize information about the observer's threshold using Bayesian inference. Confidence intervals were derived from the posterior probability distribution of the threshold parameter. Each threshold was estimated based on 30 trials, defining the minimum disparity or the minimum dot speed required for depth perception. The method uses Bayesian parameter estimation with a fine-resolution grid (70 values) to provide robust threshold estimates with confidence intervals typically ranging from ±0.1 to ±0.2 log units.

Thresholds were obtained for disparity and motion parallax cues alone, as well as for their combination. For the disparity-only condition, the individual dot displacement between the two images was manipulated, and thresholds were measured in arcmin. For the motion parallax-only condition, we manipulated the speed of the individual dots within the aperture, and thresholds were in deg/s. In the combined conditions, one cue was added to the other at its threshold value. To avoid confusion between the two combined conditions, the following terminology will be used in the rest of the article:

– **Disparity + Th_parallax_:** refers to the combined condition for which we measured a disparity threshold by manipulating the individual dot displacement between the two eyes’ images while the motion parallax value was kept constant at its threshold value (previously measured in the motion parallax-only condition) during all the trials.
– **Motion parallax + Th_disparity_:** refers to the combined condition for which we measured a motion parallax threshold by manipulating the speed of the individual dots within the aperture while the disparity value was kept constant at its threshold value (previously measured in the disparity-only condition) during all the trials.

Furthermore, for control participants, thresholds were assessed with foveal vision and with simulated scotomas of 4°, 6°, and 8° diameter. The simulated scotomas were circles displayed at the center of the stimulus window on both screens and were the same luminance as the background. The participants with MD performed the experiment with their natural scotoma and pursued the fixation cross with their PRL.

Prior to data collection, all participants were trained with disparity and motion parallax alone. Training length varied from one participant to another, as some observers needed more time to familiarize themselves with the mirror stereoscope setup and to perceive depth. Breaks were offered approximately every 10 to 15 minutes to avoid fatigue, especially in older participants.

Finally, before starting the measurements, every four threshold measurements, and after each break, the eye tracker (Eyelink 1000, SR Research; sampling rate: 1000 Hz) was calibrated for the eye with the better visual acuity, or for the dominant eye when visual acuity was similar in both eyes, using a 5-point calibration. Participants were asked to fixate each of the 5 points with their fovea or PRL. When calibration could not be completed with 5 points, we used a 3-point calibration. Calibration accuracy was verified using a validation procedure where we considered the calibration acceptable when the maximum error was below 1.0°.

### Data analysis

#### Threshold conversion

To enable direct comparison between binocular disparity and motion parallax thresholds, both measures were converted to equivalent depth difference in centimeters.

##### Disparity equivalent conversion

Binocular disparity thresholds (*δ*_d_ in arcmin) were converted to physical depth difference (Δ*d*) using a standard geometric model (Howard & Rogers, 1995). Given our viewing distance *f* = 68.5 cm and interpupillary distance (IPD) = 6.5 cm, we calculated the reference angle θ = tan⁻¹[(IPD/2)/*f*]). For each measured threshold *δ*_d_, we computed the corresponding angle in degrees as β = θ – (*δ*_d_/60), where *δ*_d_/60 converts arcminutes to degrees. The physical depth difference Δ*d* was then calculated as Δ*d* = (IPD/2)/tan(β) – *f*. Note that IPD varies from about 50 to 75 mm for most adults (Dodgson, 2004), but the magnitude of the variation is too small to account for the difference between measured disparity and motion parallax.

##### Motion parallax conversion

Motion parallax thresholds (*δ*_m_ in deg/s) were converted to equivalent depth differences using the motion/pursuit law (Nawrot & Stroyan, 2009): *d*/*f* = (retinal velocity/(pursuit velocity), where *d* represents the depth difference relative to the fixation cross, and *f* is the viewing distance (68.5 cm). The motion/pursuit ratio was calculated by dividing the retinal velocity (corresponding to the measured threshold *δ*_m_) by the pursuit velocity (3°/s during accurate pursuit). This gave depth differences as Δ*d* = (*δ*_m_/3) × *f*.

#### Statistical analysis

##### Normality testing and statistical approach

Shapiro–Wilk tests revealed nonnormal threshold distributions in both groups; therefore, depth thresholds were log-transformed prior to statistical analysis. This transformation improved the normality of residuals and homogeneity of variance. All statistical analyses were conducted on log-transformed data, with the results back-transformed to original units for interpretation and reporting.

##### Clinical subgroup classification

Participants with MD were categorized into clinically meaningful subgroups based on scotoma characteristics to examine differential patterns of cue combination within homogeneous clinical profiles:

– **Large stereo and binocular scotoma (MD1 to MD4):** MD participants with stereo scotoma > 15° in any dimension and binocular scotoma > 6° in any dimension.
– **Large stereo scotoma, small binocular scotoma (MD5):** MD participants with stereo scotoma > 15° but binocular scotoma < 6°.
– **Small or no scotomas with preserved binocular fields (MD6 to MD9):** MD participants with stereo scotoma < 15° and no binocular scotoma or relative scotomas only.
– **Compromised binocular function (MD10–MD11):** MD participants with amblyopia (MD10) or blind in one eye (MD11).

Note that MD11 was excluded from all group-level analyses involving disparity conditions due to monocularity, resulting in *n* = 10 MD participants for these analyses.

#### Control group analysis

For control participants, we conducted repeated-measures analyses of variance (ANOVAs) on log-transformed depth thresholds (in centimeters) with the condition (disparity or motion parallax), the combination (cue alone or cues combined), and the scotoma size (0°, 4°, 6°, and 8° of diameter) as within-subjects factors. Mauchly's test assessed sphericity assumptions, and Greenhouse–Geisser corrections were applied when violations occurred. Significant main effects and interactions were explored using Tukey HSD post hoc tests.

#### MD group analysis

Given the substantial interindividual variability in the clinical population, we employed a dual analytical approach for the MD group with group-level analysis and individual-level analysis.

#### Group-level analysis

*Primary analysis:* Nonparametric Friedman tests assessed overall condition effects across the four experimental conditions (disparity alone, motion parallax alone, disparity + Th_parallax_, and parallax + Th_disparity_) using *n* = 10 participants with MD (MD11 excluded due to monocularity).

*Post* *hoc comparisons:* Wilcoxon signed-rank tests were used for pairwise comparisons with Holm correction for multiple testing.

#### Optimal cue combination

To test whether depth perception follows optimal cue integration principles, we computed theoretical predictions based on the measured individual thresholds for disparity alone and motion parallax alone, as well as compared them with the experimentally measured combined thresholds (disparity + Th_parallax_ and parallax + Th_disparity_). We also compared this cue integration model to a winner-take-all model (described below), which assumes that observers rely on the more reliable single cue.

##### Variance estimation

Psychophysical thresholds were measured at 75% correct detection. To derive the underlying perceptual variance (*σ*²) from the measured thresholds, we applied a correction factor based on signal detection theory. Specifically, the relationship between the 75% threshold and the true perceptual standard deviation follows:

(1) $$\begin{array}{c} \sigma =\mathrm{threshol}d_{75}\%/0.674\; \end{array}$$

where 0.674 corresponds to the 75th percentile of the standard normal distribution. This approach provides a theoretical estimate of *σ* without requiring full psychometric function fitting, which would be less reliable given our limited trial count (30 trials per threshold) and occasional ceiling performance. The perceptual variance was then calculated as *σ*².

##### Optimal cue integration framework

According to optimal cue integration theory, when two sensory cues provide independent estimates of the same environmental property, the optimal strategy is to weight each cue by its reliability (inverse variance) to minimize the variance of the combined estimate (Ernst & Banks, 2002; Landy et al., 1995). For motion parallax (m) and disparity (d) cues, the combined variance *σ*²_combined_ is given by

(2) $$\begin{array}{c} \sigma_{{}_{\mathrm{combined}}}^{2}=\left(\sigma_{m}^{2}\times \sigma_{d}^{2}\right)/\left(\sigma_{m}^{2}+\sigma_{d}^{2}\right)\; \end{array}$$

where σ²_m_ and σ²_d_ are the variances of motion parallax and disparity thresholds measured alone, respectively.

##### Cue weight calculation

The optimal weight for each cue is proportional to its reliability:

(3) $$\begin{array}{c} w_{m}=\left(\sigma_{d}^{2}\right)/\left(\sigma_{m}^{2}+\sigma_{d}^{2}\right)\; \end{array}$$

(4) $$\begin{array}{c} w_{d}=\left(\sigma_{m}^{2}\right)/\left(\sigma_{m}^{2}+\sigma_{d}^{2}\right)\; \end{array}$$

##### Theoretical predictions for combined conditions

Our protocol measured thresholds for four conditions:

– S_d_: disparity threshold
– S_m_: motion parallax threshold
– S_d__+__m_: disparity threshold with motion parallax held constant at threshold S_m_
– S_m__+__d_: motion parallax threshold with disparity held constant at threshold S_d_

Under optimal cue integration, the weighted combination of the cues divided by their summed variance should be 0.674 at a threshold of 75% correct. Therefore,

(5) $$\begin{array}{c} \left(w_{m}S_{m}+w_{d}S_{d}\right)=0.674\times \sqrt{\sigma_{\mathrm{combined}}^{2}}\; \end{array}$$

Note that Equation 5 is a linear constraint imposed by our experimental design, in which one depth cue is fixed at its threshold level while the other cue is scaled, rather than a general property of cue integration. From this constraint, we derived the theoretical predictions:

For disparity threshold with motion parallax at threshold level (disparity + Th_parallax_):

(6) $$\begin{array}{ccc} & & \;S_{d+m,\;\mathrm{predicted}}=\left(0.674\times \sqrt{\sigma_{\mathrm{combined}}^{2}}-w_{m}S_{m}\right)/w_{d}\; \end{array}$$

For motion parallax threshold with disparity at threshold level (parallax + Th_disparity_):

(7) $$\begin{array}{ccc} & & \;S_{m+d,\;\mathrm{predicted}}=\left(0.674\times \sqrt{\sigma_{\mathrm{combined}}^{2}}-w_{d}S_{d}\right)/w_{m}\; \end{array}$$

##### Integration benefits assessment

Cue integration was assessed following the methodological recommendations of Scheller and Nardini (2024) and using Wilcoxon signed-rank tests to compare the following:

1. *Integration test:* combined thresholds against individually determined better single-cue thresholds to assess whether the cue combination provides a benefit.
2. *Optimality test:* observed combined thresholds against theoretical optimal integration predictions to evaluate integration efficiency.

##### Data exclusion criteria

Control participants were excluded from specific analyses based on condition-specific criteria to ensure data quality while preserving maximum sample size. For each combined-cue condition and each scotoma size separately, we excluded participants whose predicted thresholds were (a) negative, (b) greater than 10 cm, or (c) less than 1 × 10⁻⁴ cm. Negative predicted thresholds occurred for seven values across all participants and conditions, and they happened among participants with the largest difference between their disparity and motion parallax thresholds (e.g., perfect performance for one cue and 6.5 cm for the other one). Extreme values (greater than 10 cm or less than 1 × 10⁻⁴ cm) were due to either perfect or near-perfect performance in a single cue condition and/or a very low threshold for one single cue condition and very high for the other cue. Between 0 and 6 participants were excluded (6 for the parallax + Th_disparity_ condition with 0° scotoma because of perfect performance; average across all conditions = 2.25 participants excluded). This approach allowed participants to contribute to some analyses while being excluded from others in which their data violated model assumptions, thereby maximizing statistical power while maintaining data integrity.

##### Individual-level analysis in the MD participant group

For each participant with MD, individual cue integration patterns were examined by comparing their measured combined thresholds to both their better individual cue and maximum likelihood estimation (MLE) predictions. MD participants were then grouped by clinical characteristics (stereo and binocular scotoma sizes) to identify patterns of integration efficiency within homogeneous clinical profiles.

#### Winner-take-all

Because disparity thresholds are often substantially lower than motion parallax thresholds for control participants and could be very different for participants with MD, the combined performance might, in some cases, simply correspond to the better single-cue threshold rather than to an optimal integration of both cues. To assess whether depth thresholds in the combined cue conditions reflected genuine cue integration or a winner-take-all model, we compared the thresholds predicted by our cue integration model to a winner-take-all prediction, defined as the lower single-cue threshold. This was done for each participant and condition. For both groups of participants, we calculated the absolute errors between each model's prediction and the measured combined threshold, and we used paired Wilcoxon signed-rank tests to assess which model better matched the empirical data. Additionally, for each condition, we counted the number of participants whose combined thresholds were better predicted by the cue integration model versus the winner-take-all model.

#### Correlations between clinical characteristics and performance

##### Correlation analysis

Spearman correlations examined relationships between depth thresholds and clinical variables, including age, stereoacuity, PRL eccentricity, stereo scotoma dimensions (horizontal, vertical, and maximal extent), and binocular scotoma dimensions (horizontal, vertical, and maximal extent). The *p* values were adjusted using Benjamin–Hochberg correction for multiple comparisons.

#### Smooth pursuit analysis

##### Data processing

Eye movement data were analyzed using MATLAB (R2020b). For each trial, periods corresponding to saccades, blinks, and fixations were excluded to isolate smooth pursuit segments. Eye velocity was computed from horizontal eye position data and filtered using a zero-phase Butterworth low-pass filter (cutoff frequency: 30 Hz). Each trial was divided into two phases, initial and return, corresponding to the direction of the target motion (the fixation cross) before and after the mid-trial reversal. For each phase, pursuit gain (eye velocity/target velocity) was computed during smooth pursuit periods. Trials with insufficient data or extreme outliers were discarded. Gain values close to 1.0 indicate accurate velocity matching, while values consistently below 1.0 suggest impaired smooth pursuit ability. Additionally, smooth pursuit onset latency and latency following the direction reversal were computed by detecting significant deviations in eye velocity above zero baseline (corresponding to the stationary eye state before pursuit initiation). We also extracted the number of saccades within each trial, as well as their timing relative to the target motion. Summary metrics (including pursuit gain, latency, and number of saccades) were computed for each trial and averaged across conditions for statistical analyses (motion parallax, cues combined or not, or each scotoma size).

##### Correlations with pursuit metrics

Correlations between pursuit performance (gain, number of saccades) and depth thresholds across all experimental conditions were assessed using Spearman correlations.

#### Statistical considerations

##### Multiple comparisons

All analyses involving multiple comparisons applied appropriate corrections (Bonferroni, Holm, Benjamin–Hochberg, or Tukey as specified).

##### Effect sizes

Effect sizes are reported as partial $\eta_{p}^{2}$ for ANOVAs, Cohen's *d* for *t* tests, *r* = *Z*/√*N* for Wilcoxon signed-rank tests, Kendall's W for Friedman tests, and Spearman's ρ for correlations.

## Results

The main objective of this study was to assess whether combining disparity and motion parallax improves depth perception in participants with central field loss. MD and control participants showed markedly different response patterns to cue combination. Therefore, we first present detailed analyses of MD performance with reference to control results used as a benchmark, followed by a detailed presentation of the control participants’ results.

### MD participants show variable depth perception deficits

We first focused on evaluating depth perception in participants with macular degeneration across the four conditions (disparity alone, motion parallax alone, disparity + Th_parallax_, and motion parallax + Th_disparity_) at the group level using a Friedman test on the log-transformed thresholds. Note that the results of participant MD11 were discarded from the group analysis due to missing data as he was blind in one eye and could not perceive disparity.

**Figure 3.**
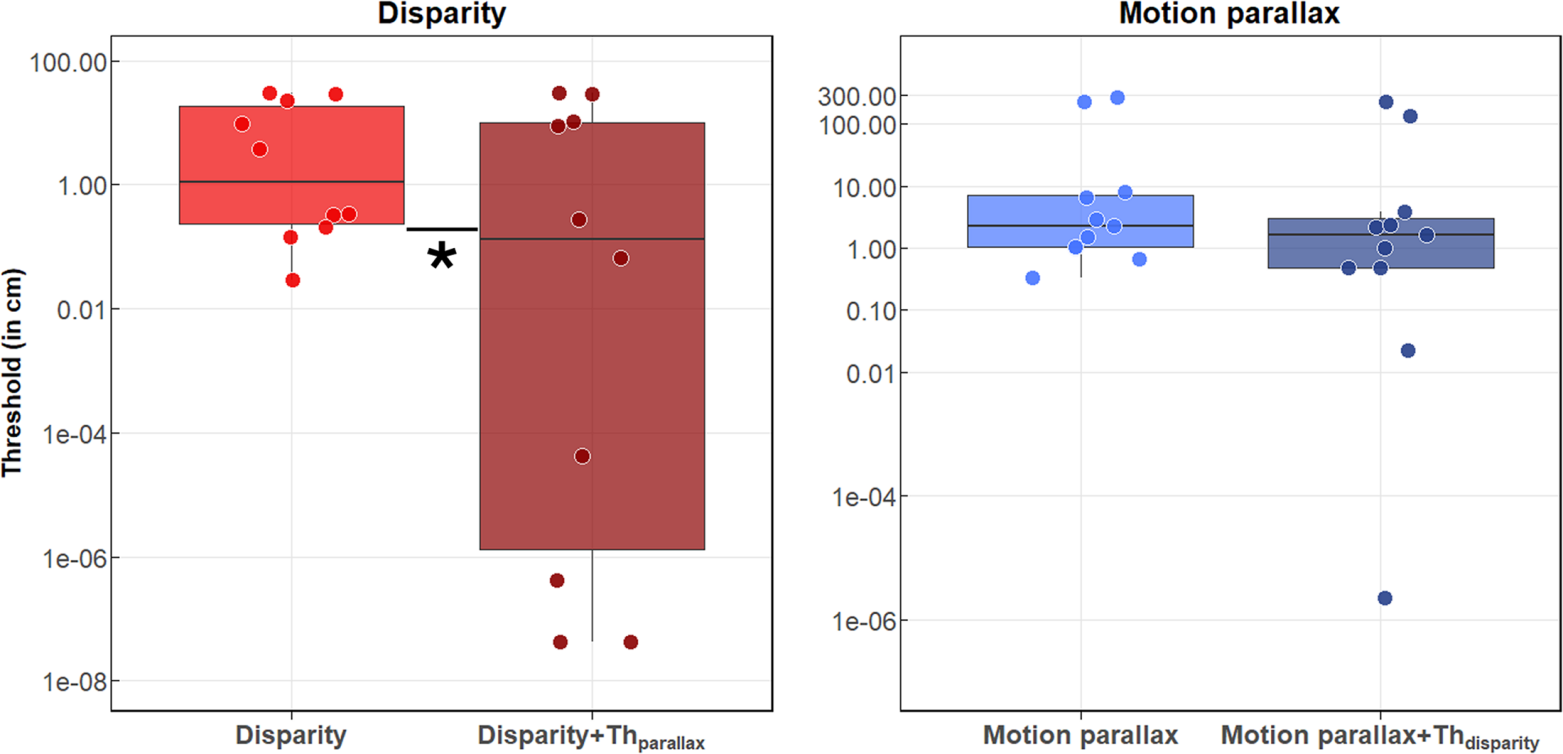
Depth thresholds (in centimeters) measured for disparity (red) and motion parallax (blue) in participants with MD. Light boxes show the threshold for the cue alone, while dark boxes show thresholds in the combined conditions. The colored dots represent the individual depth thresholds for each MD participant. Each boxplot shows the median threshold (dark internal horizontal line) and the first and third quartiles (top and bottom edges of the box). Note that disparity and motion parallax have different scales. *p < 0.05.

The test revealed a significant effect of the condition on measured depth thresholds among participants with macular degeneration, *χ*²(3) = 11.02, *p* = 0.012, with a large effect size (Kendall's *W* = 0.367). Post hoc pairwise comparisons using the Wilcoxon signed-rank test (Holm-adjusted) showed a significant difference between thresholds measured for disparity alone and disparity + Th_parallax_ (*p* = 0.036, *r* = 0.870), with lower thresholds for the combined condition. No other pairwise comparisons reached statistical significance after correction (all adjusted, *p* > 0.05). Results are presented in Figure 3.

Crucially, while median [Q25–Q75] thresholds were relatively low (2.05 [0.239–19.1] cm for disparity, 2.28 [1.03–7.23] cm for motion parallax, 0.167 [1.08e-5–9.95] cm for disparity + Th_parallax_, and 1.65 [0.482–3.11] cm motion parallax + Th_disparity_), the interquartile ranges were extremely large, highlighting the considerable variability in performance across MD participants. This variability in MD participants contrasts with the pattern observed in control participants who had lower measured thresholds for both cues compared to MD participants and were more consistent as a group (e.g., median thresholds for controls without scotoma ranging from 0.0001 [0.00002–0.076] cm for the combined condition motion parallax + Th_disparity_ to 0.149 [0.083–0.347] cm for motion parallax; further details are provided in Section 5 of the Results). Moreover, it is worth mentioning that MD11 (blind in one eye) was still able to perceive depth from motion parallax, and his measured threshold was 2.85 cm, which is around the median value for the group in the motion parallax-only condition.

Because of this large interindividual variability, far exceeding that seen in controls, group-level statistics may obscure meaningful individual differences. We therefore next examined how disparity and motion parallax were combined at the group level before turning to individual cue integration analyses to better capture this variability.

### Cue integration efficiency varies by clinical subgroup

Having established that depth thresholds were better in the disparity + Th_parallax_ condition compared to the disparity-alone condition at the group level but that the variability was very large, we then explored more finely whether MD participants were able to combine disparity and motion parallax in a way consistent with optimal cue integration. Following Scheller and Nardini (2024), we conducted two key statistical tests using Wilcoxon signed-rank tests: (a) *integration test*, in which we compared combined thresholds against the individually determined better single-cue thresholds to assess whether cue combination provides benefit, and (b) *optimality test*, in which we compared the combined thresholds against theoretical optimal integration predictions to evaluate integration efficiency.

**Figure 4.**
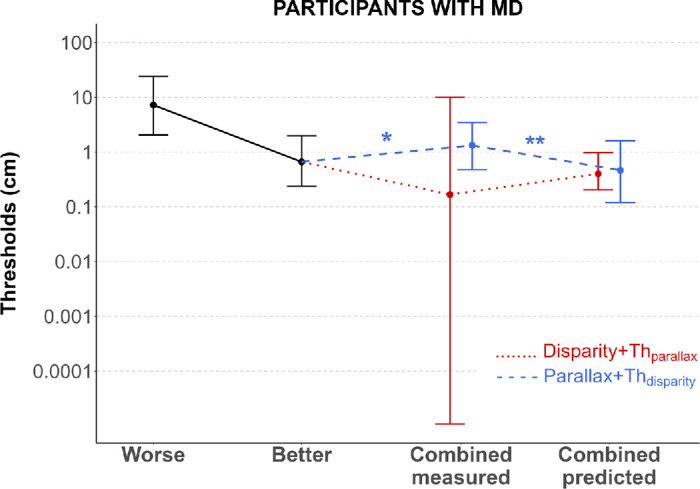
Cue integration at the group level for participants with MD. Each dot corresponds to the median thresholds with the associated 25th quartile and 75th quartile, for the worse individual cue, the better individual cue, both cues, and the MLE predictions. Both cues correspond to the two combined conditions, with disparity + Th_parallax_ in red and parallax + Th_disparity_ in blue. *p < 0.05, **p < 0.01.

When comparing thresholds for the disparity + Th_parallax_ condition to the better individual cue, group analysis using Wilcoxon signed-rank tests showed no significant cue integration (*p* = 0.375) in this combined condition. This combined condition (median disparity + Th_parallax_ = 0.167 [1.08e-5–9.95] cm) did not provide any advantage over the better individual cue performance (median better cue = 0.662 [0.239–1.977] cm), contrasting sharply with the significant integration benefit observed in controls (see Results, Control participants: validation of experimental paradigm). Importantly, the combined condition disparity + Th_parallax_ matched theoretical optimal predictions (*p* = 0.846), with predicted thresholds (median = 0.40 [0.204–0.983] cm) not significantly different from observed values (see Figure 4). This suggests that, while MD participants did not benefit at a group level from the combination of the two cues, their performance was actually consistent with what would be expected from optimal combination theory. This could be because, in our optimal cue integration model, there is no benefit if one cue is much more sensitive than the other. We will explore this possibility in the following sections. Again, these results contrast with those found in the control group, for which we observed optimal integration, or even better than predicted integration, for the three smallest simulated scotoma sizes.

Furthermore, for the combined condition parallax + Th_disparity_, MD participants showed significant performance degradation (*p* = 0.033) rather than integration benefit. Thresholds measured in this combined condition (median = 1.323 [0.475–3.475] cm) were significantly worse than for the better individual cue (median = 0.662 [0.239–1.977] cm) at the group level. Critically, combined thresholds significantly deviated from the theoretical predictions (*p* = 0.010), with observed thresholds substantially worse than predicted optimal values (median predicted = 0.465 [0.120–1.608] cm), indicating suboptimal integration. In comparison, no significant degradation or suboptimal integration was observed in the control group.

These results suggest that cue integration in MD participants at the group level depends on the primary depth cue used during the measurement and may exhibit suboptimal characteristics when disparity is set at its threshold value. Importantly, as was mentioned previously, MD participants exhibited very large variability in integration performance, prompting us to look more closely at individual results.

**Figure 5.**
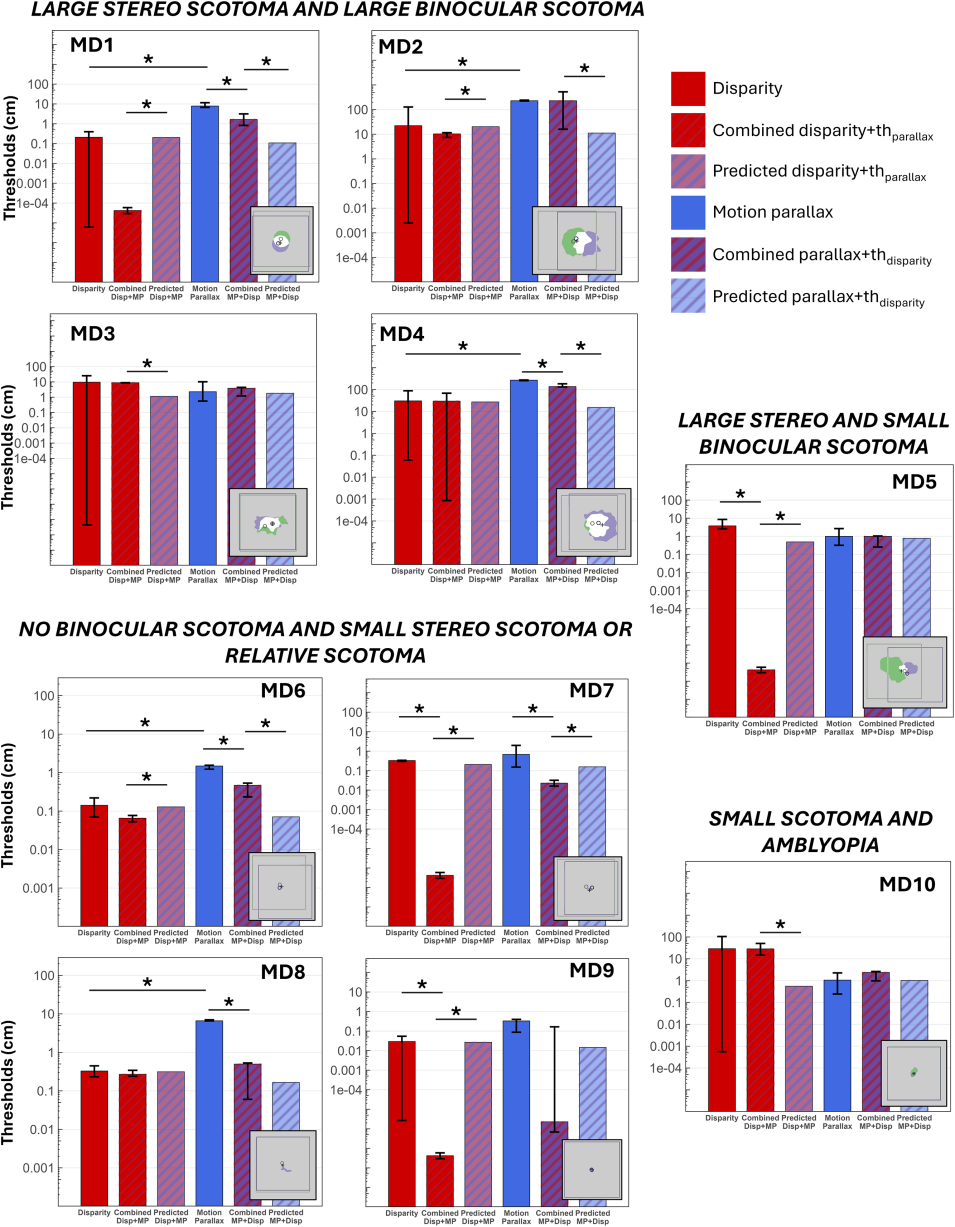
Individual thresholds for each individual with MD, divided into clinical subgroups. Each plot shows from left to right the threshold for disparity alone, measured disparity + Th_parallax_, predicted disparity + Th_parallax_, motion parallax alone, measured parallax + Th_disparity_, and predicted parallax + Th_disparity_. Bars represent 95% confidence intervals. Stars indicate a significant difference between two thresholds (i.e., no overlap between 95% confidence intervals). The inset within each panel represents the binocular and stereo scotomas for that individual, as described in Figure 1.

For this reason, we grouped MD participants based on their clinical characteristics and, more specifically, their stereo and binocular scotoma sizes. Each individual's results are detailed in Figure 5, which shows the measured threshold for disparity alone and disparity + Th_parallax_, the predicted threshold for disparity + Th_parallax_, the measured threshold for motion parallax alone and parallax + Th_disparity_, and the predicted threshold for parallax + Th_disparity_. For every participant except MD3, MD5, and MD10, the worse single cue corresponds to the motion parallax threshold, in line with previous studies that show that this cue is typically less sensitive for depth perception than disparity in normal vision (Hartle & Wilcox, 2021; McKee & Taylor, 2010; Rogers & Graham, 1982), suggesting that this hierarchy is preserved in most MD participants despite their central field loss. Each threshold is shown with its associated 95% confidence interval (CI). Thresholds were considered significantly different when their respective CIs did not overlap.

*MD participants with large stereo scotomas and large binocular scotomas (MD1 to MD4):* Results show that, in this subgroup, disparity thresholds were comparable to thresholds measured for the disparity + Th_parallax_ condition for every participant, meaning that adding motion parallax at its threshold value has little to no impact. Note that for MD1, the CI for the disparity-alone threshold was very large and overlapped with the CI of the combined measured threshold, which is why we did not consider the difference significant. However, adding disparity at its threshold value to motion parallax (parallax + Th_disparity_ condition) had more impact on the measured combined threshold for MD1 and MD4, who had significantly lower thresholds in the combined condition, while no difference was found for MD2 and MD3. We then compared the measured thresholds in the combined conditions with the predicted thresholds to determine if optimal integration was achieved. For the disparity + Th_parallax_ condition, MD1 and MD2 had better than predicted thresholds in the combined condition, and MD4 had optimal integration (combined threshold comparable to MLE prediction), but MD3 exhibited suboptimal integration. In the parallax + Th_disparity_ condition, MD1, MD2, and MD4 had significantly worse measured thresholds compared to the MLE prediction (suboptimal integration), but no difference was found for MD3 (optimal integration). Overall, the results in this subgroup were mixed, with different patterns of results in the parallax + Th_disparity_ condition compared to the disparity + Th_parallax_ condition. We also note that for some participants, no difference was found between the threshold for a cue presented alone or in a combined condition, while at the same time, a significant difference between this combined threshold and the predicted threshold was observed. This may indicate that an alternative model could better predict patients’ thresholds, like the winner-take-all model that we will develop in the next results section.

*MD participant with large stereo scotomas and small binocular scotomas (MD5):* MD5 presented a clear benefit of combining motion parallax to disparity (disparity + Th_parallax_ condition), with a significantly smaller threshold measured in this combined condition compared to the disparity cue presented alone and compared to the predicted threshold (optimal integration). Motion parallax threshold and parallax + Th_disparity_ threshold were very similar, and no difference was found between this measured combined threshold and the MLE prediction.

*MD participants with no binocular scotoma and small stereo scotoma (MD6 to MD9):* As previously mentioned, MD participants in this group had, on average, thresholds similar to those measured in the control group. Similarly, cue integration patterns looked like those of controls. MD7 and MD9 presented a much lower threshold for the combined condition disparity + Th_parallax_ compared to disparity alone, while no difference was observed for MD6 and MD8. MD6, MD7, and MD8 also had lower thresholds in the parallax + Th_disparity_ condition, compared to motion parallax presented alone. Moreover, MD6, MD7, and MD8 also had better than predicted thresholds in the disparity + Th_parallax_ condition, while results for the parallax + Th_disparity_ condition were more mixed. MD7 had a better than predicted threshold for the parallax + Th_disparity_ condition, while MD8 and MD9 demonstrated optimal integration, and MD6 demonstrated suboptimal integration. This seems to indicate that with a small stereo scotoma, participants with MD benefit from combining both cues, particularly when disparity is added to threshold parallax.

*MD participants with small scotomas but no binocular combination* *,* *either due to amblyopia with strabismus (MD10) or blindness in one eye (MD11):* No threshold for disparity was measured for MD11, so we will only discuss MD10 results. As previously mentioned, his amblyopia prevented him from perceiving fine depth from the disparity cue. Thus, as expected, the measured thresholds did not improve when both cues were presented compared to each cue presented alone. The combined measured threshold in the disparity + Th_parallax_ condition was even significantly worse than the MLE prediction, confirming suboptimal integration.

These contrasting patterns of results between subgroups strongly suggest that the scotoma characteristics are key determinants of performance and cue combination benefits. Before testing this hypothesis quantitatively through correlations with clinical variables, we first assessed whether the combined performance reflected genuine cue integration rather than a winner-take-all strategy, where participants simply relied on their better single cue.

### Winner-take-all model in participants with MD

Because disparity thresholds and motion parallax thresholds were vastly different for many participants with MD, their combined performance might in some cases simply correspond to the better single-cue threshold rather than to an optimal integration of both cues. To assess whether this was the case or not, we compared our predicted thresholds with thresholds predicted by the winner-take-all model, which simply corresponds to the lower single-cue threshold. Results are reported in Table 2.

**Table 2.** Comparison of the cue integration and the winner-take-all models for participants with MD. *Note*: The first part of the table presents group results. For each combined condition (disparity + Th_parallax_ and parallax + Th_disparity_), we report the model that predicts the most participants’ thresholds (and the number out of 10), the better model based on the Wilcoxon test and the associated *p* value, and the median absolute errors between the measured thresholds and the predicted thresholds for each model with the corresponding 25th quartile and 75th quartile. The second part of the table presents individual results for each patient. The better individual model corresponds to the model that predicted the threshold closest to the measured combined threshold. Absolute errors between each model and the measured combined thresholds are also reported for each MD participant.

Group results: participants with MD					
				Absolute errors between measured threshold and model, median (Q25–Q75), cm	
Condition	Model that predicts the most participants’ thresholds		Difference between models (Wilcoxon) and better model	Integration	Winner-take-all
Disparity + Th_parallax_	Cue integration (7/10)		No difference (*p* = 0.77)	0.348 (0.098–6.413)	0.543 (0.109–5.173)
Parallax + Th_disparity_	Winner-take-all (8/10)		Winner-take-all (*p* = 0.03)	0.879 (0.254–1.913)	0.825 (0.203–1.528)
Individual results: participants with MD					

			Absolute errors between measured threshold and model, median (Q25–Q75), cm		
Condition	Participant	Better individual model	Cue integration	Winner-take-all	

Disparity + Th_parallax_	MD1	Cue integration	0.203	0.209	
	MD2	Cue integration	9.921	11.994	
	MD3	Winner-take-all	7.723	6.566	
	MD4	Winner-take-all	2.484	0.756	
	MD5	Cue integration	0.488	0.993	
	MD6	Cue integration	0.063	0.076	
	MD7	Cue integration	0.208	0.331	
	MD8	Cue integration	0.043	0.059	
	MD9	Cue integration	0.027	0.029	
	MD10	Winner-take-all	27.933	27.427	
Parallax + Th_disparity_	MD1	Winner-take-all	1.546	1.445	
	MD2	Winner-take-all	217.205	206.024	
	MD3	Winner-take-all	2.035	1.556	
	MD4	Winner-take-all	121.871	106.647	
	MD5	Winner-take-all	0.228	0	
	MD6	Winner-take-all	0.397	0.326	
	MD7	Cue integration	0.134	0.308	
	MD8	Winner-take-all	0.331	0.168	
	MD9	Cue integration	0.015	0.029	
	MD10	Winner-take-all	1.362	1.324	

Analysis using Wilcoxon signed-rank tests revealed a different pattern of results for the two conditions: disparity + Th_parallax_ and parallax + Th_disparity_. In the disparity + Th_parallax_ condition, the difference between the two models was not significant at the group level (*p* > 0.05). However, when looking at individual results, for 7 out of the 10 patients, the cue integration model better predicted the measured combined threshold. Conversely, in the parallax + Th_disparity_ condition, the two models were significantly different (*p* = 0.037), and the winner-take-all was the better model. This was confirmed by the individual results: 8 out of 10 patients’ performance was better predicted by the winner-take-all model.

These analyses revealed that, while the performance of most MD participants was consistent with cue integration in the disparity + Th_parallax_ condition, most appeared to rely primarily on their better single cue when disparity at its threshold value was added to motion parallax (parallax + Th_disparity_ condition). The variability in integration patterns across participants reinforces the need to identify which clinical characteristics predict successful cue combination. We therefore examined correlations between scotoma measurements and depth perception thresholds.

### Scotoma size strongly correlates with disparity but not motion parallax performance

We performed Spearman correlations between MD participants’ scotoma measurements (horizontal and vertical maximal dimensions of the stereo scotoma and the binocular scotoma) and their depth perception thresholds across all conditions. We also explored correlations with age, stereoacuity (in arcsec, assessed using the Random Dot Stereo Butterfly Test), and PRL eccentricity.

**Figure 6.**
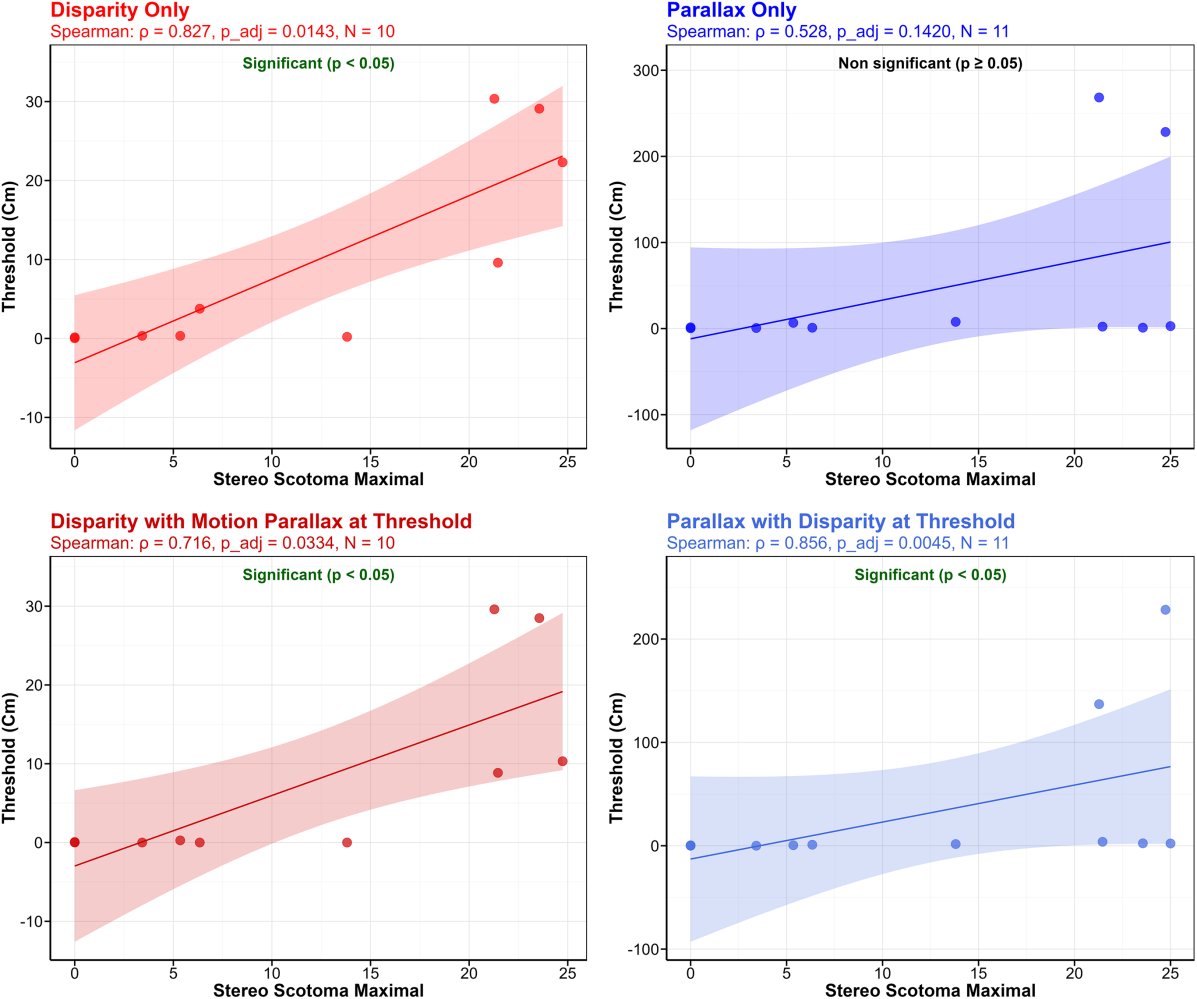
Correlations between measured thresholds for each condition and the maximum dimension of the stereo scotoma size in MD participants.

Disparity-based conditions showed strong correlation with stereo scotoma size, while motion parallax presented alone remained largely unrelated to central field loss (see Figure 6). More precisely, for the disparity-only condition, significant positive correlations were observed between the thresholds and all stereo scotoma measurements (horizontal: ρ = 0.802, *p* = 0.016; vertical: ρ = 0.813, *p* = 0.012; maximal: ρ = 0.827, *p* = 0.014). For the binocular scotoma, only the vertical dimension showed a significant correlation with the disparity threshold (vertical: ρ = 0.705, *p* = 0.041), while other dimensions approached but did not reach significance (horizontal extent: ρ = 0.653, *p* = 0.052; maximal extent ρ = 0.653, *p* = 0.052). As might be expected, stereoacuity correlated significantly with disparity thresholds (ρ = 0.784, *p* = 0.016). Crucially, motion parallax alone showed no significant correlation with any clinical characteristics (all *p* > 0.05), suggesting that this monocular cue remains relatively preserved, even with extensive central field loss.

Both combined-cue conditions showed similar correlation patterns to disparity alone, with all stereo scotoma measurements significantly correlating with thresholds (disparity + Th_parallax_: all adjusted *p* < 0.05; parallax + Th_disparity_: all adjusted *p* < 0.01). Additional correlations emerged for PRL eccentricity in the disparity + Th_parallax_ condition (ρ = 0.684, *p* = 0.038) and stereo acuity in the parallax + Th_disparity_ condition (ρ = 0.731, *p* = 0.014).

All together, these results confirm that the extent of the scotoma, particularly the stereo scotoma, is the most correlated individual characteristic with the performance in the depth discrimination task. This is true for every condition that involves disparity, but not for motion parallax presented alone.

### Smooth pursuit

Another factor that could impact the measured motion parallax thresholds is the ability to maintain accurate smooth pursuit. In fact, depth from motion parallax is provided by the relative motion of the dots within the moving window, and accurate pursuit ensures the participant is tracking this moving window effectively. However, previous studies showed that individuals with macular degeneration have reduced pursuit gain, increased variability (Shanidze, Fusco, Potapchuk, Heinen, & Verghese, 2016), and more saccades (Shanidze, Lively, Lee, & Verghese, 2022). We wanted to see if this was also the case in our experiment and if this was correlated with MD participants’ thresholds.

**Figure 7.**
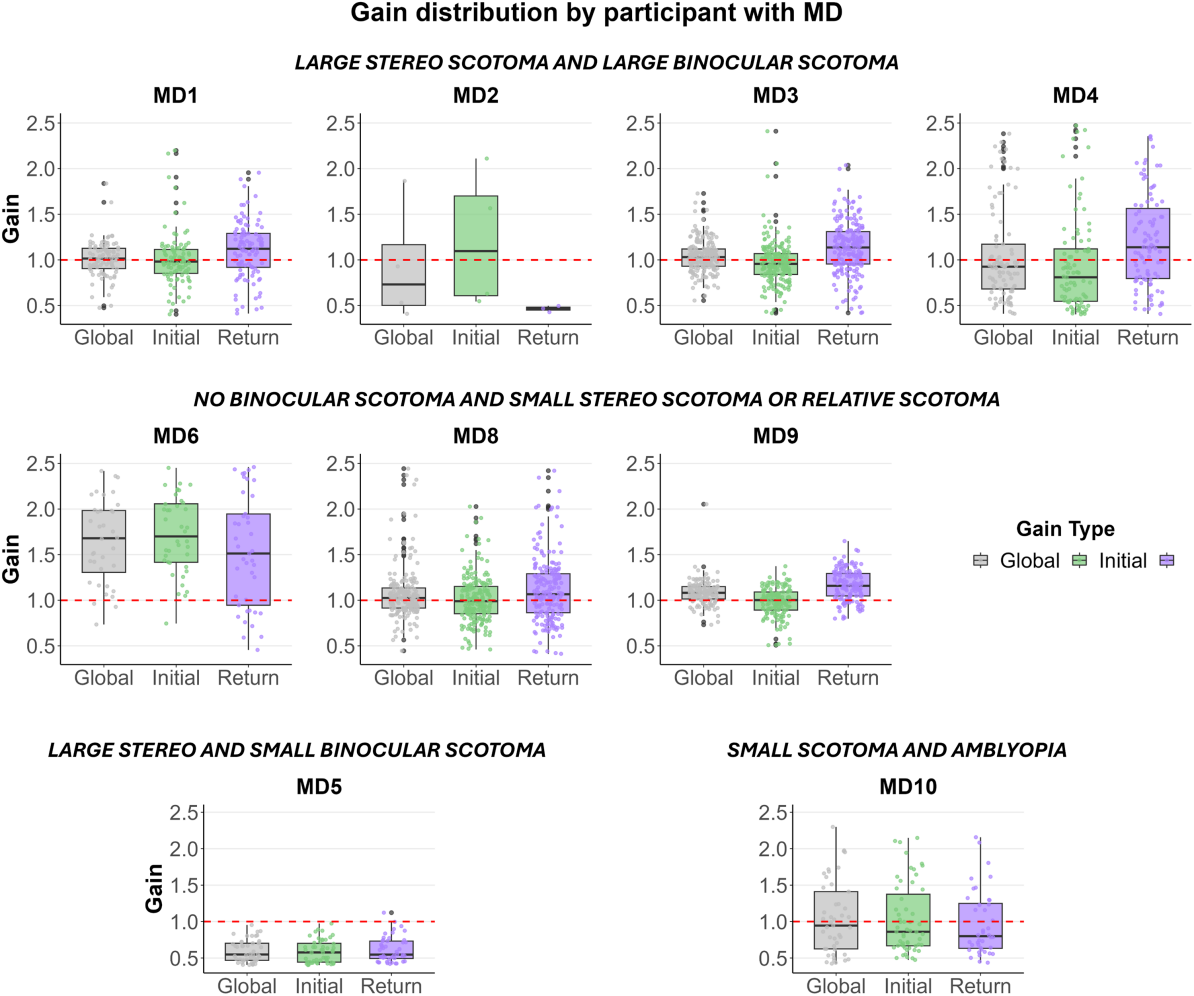
Pursuit gains measured for each individual with MD. Each individual dot represents a trial. Gray data show pursuit gains during the whole trials. Green data show pursuit gains during the initial phase of pursuit (before direction reversal). Purple data represent pursuit gains during the return phase (after direction reversal).

At the group level, MD participants and controls had comparable pursuit gain (mean for every condition in the MD group: 1.07 ± 0.26; mean control group: 1.07 ± 0.2). However, participants with MD exhibited much larger variability for pursuit gain, ranging from a minimum average of 0.63 for MD5 to a maximum of 1.61 for MD6. Figure 7 shows pursuit gains for each participant with MD. “Global” corresponds to the pursuit gain during the entire trials, “Initial” refers to the direction of the stimulus window during the first half of the stimulus, and “Return” corresponds to the direction of the stimulus window during the second half of each trial, after the direction change. Note that pursuit gain was slightly higher during the return phase, which was due to anticipatory eye movements when the stimulus window changed direction. This was also true in the control group. Similarly, the number of saccades was more variable in the MD group, with a minimum of 2.67 saccades for MD9 to a maximum of 6.19 for MD8 on average (mean: 4.4 ± 1.2 saccades; mean for controls: 3.1 ± 2.3 saccades). Moreover, the smooth pursuit onset latency was more elevated in the MD participant group (mean: 280.02 ± 97.66 ms; mean for controls: 192.68 ± 123.36 ms), but the latency following the direction reversal was much shorter and comparable to that of controls (mean patients: 20.66 ± 10.74 ms; mean controls: 23.74 ± 12.92 ms).

Correlations were performed between thresholds measured in each condition and pursuit gains, and no significant correlation was found (all *p* > 0.05). Similarly, no significant correlation was found between the thresholds and the number of saccades (all *p* > 0.05).

### Control participants: validation of experimental paradigm

To validate our experimental approach and confirm that our depth perception paradigm produces expected results under normal viewing conditions, we also examined performance in control participants. These findings also allow us to determine whether the deficits observed in MD participants simply reflect the consequence of central field loss or indicate more complex adaptations in depth-processing mechanisms.

### Depth thresholds in normal vision and with simulated central scotomas

Statistical analysis consisted of a repeated-measures ANOVA on log-transformed depth thresholds with the condition (disparity or motion parallax), the combination (cue alone or cues combined), and the scotoma size (0°, 4°, 6°, and 8° of diameter) as within-subjects factors.

The ANOVA led to significant main effects of the condition (*F*(1, 18) = 4.17, *p* < 0.001, $\eta_{p}^{2}$ = 0.746), the combination (*F*(1, 18) = 5.89, *p* < 0.001, $\eta_{p}^{2}$ = 0.804), the scotoma size (*F*(2.28, 48.21) = 2.12, *p* = 0.013, $\eta_{p}^{2}$ = 0.188), and a significant condition × combination interaction (*F*(1, 18) = 3.22, *p* < 0.001, $\eta_{p}^{2}$ = 0.641).

### Control participants demonstrated the expected hierarchy of depth cue precision

Post hoc pairwise comparisons (Tukey adjusted) revealed that thresholds for disparity alone were significantly lower than for motion parallax alone (median disparity = 0.023 [0.015–0.033] cm; median motion parallax = 0.298 [0.199–1.335] cm; *t*(18) = 7.262, *p* < 0.001, *d* = 2.356), confirming that binocular disparity provides more precise depth information than motion parallax.

### Combining depth cues significantly improved performance

**Figure 8.**
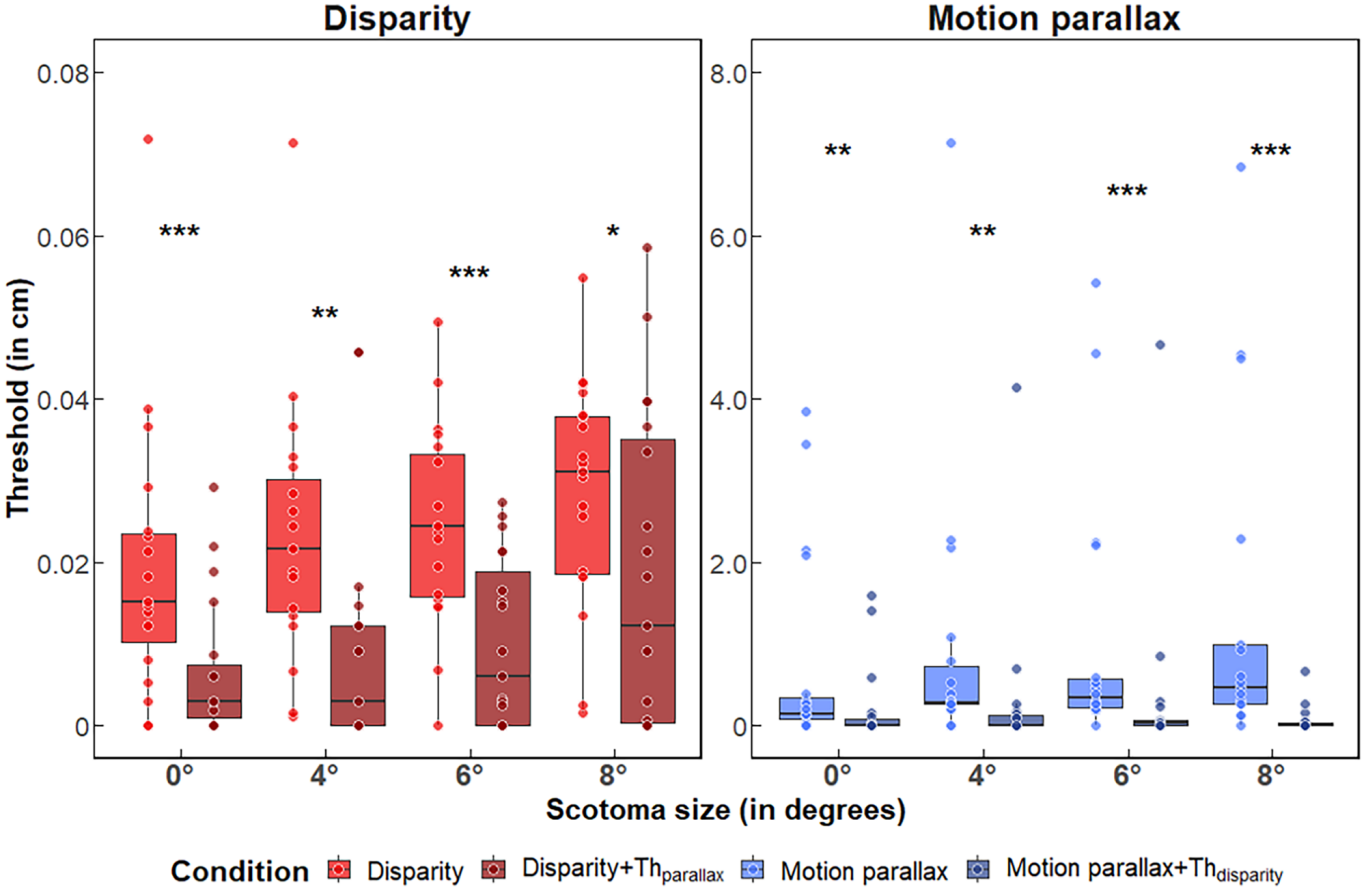
Depth thresholds in centimeters measured for disparity (red) and motion parallax (blue) in control participants. Light boxes show the threshold for the cue alone, while dark boxes show thresholds in the combined conditions. Note that disparity and motion parallax have different scales for the vertical axes. *p < 0.05, **p < 0.01, ***p < 0.001.

Analysis also shows that combining depth cues significantly improved performance across all viewing conditions. Thresholds for the disparity + Th_parallax_ condition were significantly lower than thresholds for disparity alone (median disparity + Th_parallax_ = 0.006 [0.0001–0.017] cm; *t*(18) = 5.718, *p* < 0.001, *d* = 1.855), and thresholds for the parallax + Th_disparity_ condition were significantly lower than thresholds for motion parallax alone (median parallax + Th_disparity_ = 0.226 [0.0001–0.078] cm; *t*(18) = 8.086, *p* < 0.001, *d* = 2.623). These results were consistent across all scotoma sizes, with pairwise comparisons that showed significantly reduced thresholds when the two cues were combined in each case (all *p* < 0.05), demonstrating that the benefits of cue combination persist even with simulated central scotomas up to 8° of diameter. Results are presented in Figure 8.

Critically, these results suggest that simulated central scotomas in control participants produced different patterns of impairment compared to participants with macular degeneration. Controls showed proportional increases in both disparity and motion parallax thresholds with increasing scotoma size, whereas MD participants demonstrated selective preservation of motion parallax processing regardless of scotoma extent.

### Stable integration mechanisms in controls contrast with variability among MD individuals

Following the same methodological framework used for patients, we conducted Wilcoxon signed-rank tests to assess integration benefits and integration optimality.

*Control participants demonstrated optimal or better-than-predicted cue integration:* Controls were much more sensitive to disparity than to motion parallax, which led to a consistent but asymmetric pattern of cue integration that varied systematically with scotoma size and depended critically on which depth cue served as the primary signal.

Results from the Wilcoxon signed-rank tests revealed that combining disparity with motion parallax at its threshold value (disparity + Th_parallax_ condition) led to significant cue integration across smaller scotoma sizes. Participants demonstrated improved thresholds when both cues were available compared to their individually determined better single cue (0°: *p* = 0.010; 4°: *p* = 0.036; 6°: *p* = 0.004), although this integration benefit was absent with the largest simulated scotoma (8°: *p* = 0.169). Importantly, the observed integration in this combined condition consistently deviated from theoretical combined predictions, with measured performance being significantly better than theoretical optimal values (0°: *p* = 0.045; 4°: *p* = 0.045; 6°: *p* = 0.004; but 8°: *p* = 0.241), suggesting a better than predicted integration.

**Figure 9.**
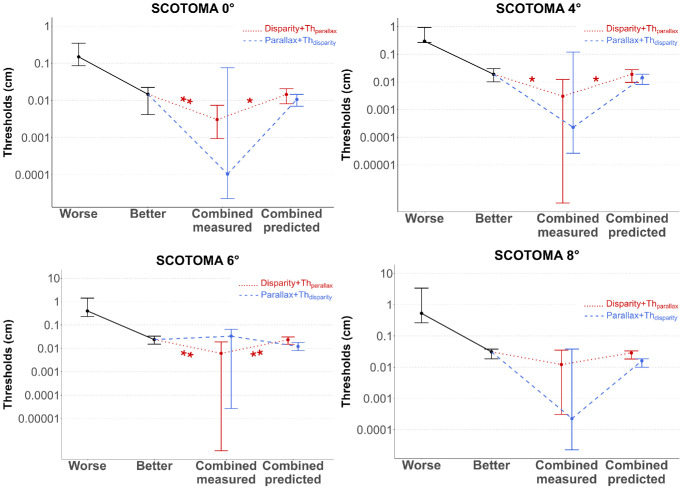
Cue integration in the control group for each simulated scotoma size. Each dot corresponds to the median thresholds with the associated Q25 and Q75 in the control group, for the worse individual cue, the better individual cue, the combined cues, and the combined predictions. The combined cues correspond to the two combined conditions, with disparity + Th_parallax_ in red and parallax + Th_disparity_ in blue. Results are shown for 0°, 4°, 6°, and 8° simulated scotoma. *p < 0.05, **p < 0.01.

In contrast, in the parallax + Th_disparity_ condition, there was no evidence of cue integration across any scotoma size. As a reminder, we now compare the combined thresholds parallax + Th_disparity_ to the better cue alone (Figure 9), which explains why this new result differs from the one presented in the previous section, where we compared thresholds for the parallax + Th_disparity_ condition to thresholds for the motion parallax–alone condition (Figure 8). Note the average combined measured thresholds in the parallax + Th_disparity_ condition are high due to larger variability than in the disparity + Th_parallax_ condition (see Figure 9). No significant difference was found between these measured thresholds and the theoretically optimal predicted thresholds, or with the better threshold (all *p* > 0.05), indicating that while integration did not occur, the lack of benefit was consistent with theoretical expectations. The combined measured thresholds were, nonetheless, significantly better (lower) than the thresholds measured for the worse cue alone.

These findings reveal a stable but asymmetric integration pattern in control participants, where cue integration depends critically on which depth cue serves as the primary signal, with integration occurring predominantly when disparity is the primary cue. Integration benefits are maintained across all small to moderate simulated scotoma sizes (0° to 6°) but fail to achieve statistical significance with the largest scotoma size (8°).

### Winner-take-all model in control participants

Because disparity thresholds were substantially lower than motion parallax thresholds for control participants, the combined performance might in some cases simply correspond to the better single-cue threshold rather than to an optimal integration of both cues. As we did with participants with MD, we compared our predicted thresholds with thresholds predicted by the winner-take-all model, which simply corresponds to the lower single-cue threshold. Results are reported in Table 3.

**Table 3.** Comparison of the cue integration and the winner-take-all models for control participants. *Note*: For each combined condition (disparity + Th_parallax_ and parallax + Th_disparity_) and each scotoma size (0°, 4°, 6°, and 8°), we report the model that predicts the most participants’ thresholds (and the number out of 19 total controls), the better model based on the Wilcoxon test and the associated *p* value, and the median absolute errors between the measured thresholds and the predicted thresholds by each model with the corresponding 25th quartile and 75th quartile.

Group results: participants with MD					
				Absolute errors between measured threshold and model, median (Q25–Q75), cm	
Condition	Scotoma size	Model that predicts the most participants’ thresholds	Difference between models (Wilcoxon) and better model	Integration	Winner-take-all
Disparity + Th_parallax_	0°	Cue integration (13/19)	Winner-take-all (*p* = 0.013)	0.0074 (0.0023–0.0151)	0.0073 (0.0061–0.0183)
	4°	Cue integration (16/19)	Cue integration (*p* < 0.001)	0.0172 (0.0107–0.0269)	0.0182 (0.0124–0.0286)
	6°	Cue integration (15/19)	Winner-take-all (*p* = 0.003)	0.0160 (0.0072–0.0262)	0.0175 (0.0073–0.0304)
	8°	Cue integration (13/19)	Cue integration (*p* = 0.020)	0.0183 (0.0106–0.0310)	0.0183 (0.0101–0.0322)
Parallax + Th_disparity_	0°	Cue integration (11/19)	Cue integration (*p* = 0.005)	0.0106 (0.0087–0.0181)	0.0232 (0.0204–0.0366)
	4°	Cue integration (10/19)	No difference (*p* = 0.188)	0.0201 (0.0111–0.0835)	0.0366 (0.0185–0.0694)
	6°	Winner-take-all (8/19)	No difference (*p* = 0.720)	0.0236 (0.0157–0.0498)	0.0364 (0.0200–0.0489)
	8°	Cue integration (10/19)	No difference (*p* = 0.120)	0.0178 (0.0134–0.0340)	0.0309 (0.0225–0.0421)

Analysis using paired Wilcoxon signed-rank tests showed a significant difference between the two models for the disparity + Th_parallax_ condition and for each scotoma size. For 0° and 6° scotoma sizes, results indicate that the winner-take-all model better predicted the measured thresholds (respectively *p* = 0.013 and *p* = 0.003). Conversely, for the 4° and 8° scotomas, the better model was the cue integration model (*p* < 0.001 and *p* = 0.02). However, it is important to note that for the 0° and 6° conditions (as well as for the 4° and 8°), even though winner-take-all was statistically the better model, most of the measured participants’ thresholds were better predicted by the cue integration model at the individual level. This different result comes from slightly larger absolute errors that could occur with the cue integration model.

For the parallax + Th_disparity_ condition, paired Wilcoxon signed-rank tests showed no significant difference between the two models for 4°, 6°, and 8° scotomas (*p* > 0.05). For the 0° scotoma, models were significantly different, and the cue integration model better explained the measured thresholds (*p* = 0.005). Moreover, as noted previously for the 0°, 4°, and 8° scotoma conditions, the individual absolute errors were smaller for the cue integration model.

Overall, these analyses revealed that control participants predominantly used cue integration rather than a winner-take-all strategy, particularly at an individual level, where the integration model better predicted most participants’ thresholds across conditions. The mixed statistical results at the group level (with winner-take-all performing better for some scotoma sizes) likely reflects the small absolute differences between models when integration is highly efficient. Critically, this consistent integration pattern, in controls, even with large simulated scotomas, contrasted sharply with the variable integration observed in participants with MD.

### Summary

These findings confirm that our experimental paradigm reliably elicits optimal cue integration in participants with normal vision. Winner-take-all analysis revealed that while controls consistently engaged in genuine cue integration, MD participants showed variable patterns that depended critically on which cue served as the primary signal. The presence of simulated scotomas alone did not disrupt the fundamental mechanisms of depth cue combination in controls. Finally, the integration impairments observed in participants with macular degeneration reflect changes beyond simple central scotomas. This is shown particularly by the preserved integration capabilities in controls with simulated scotomas, contrasted with the highly variable and often suboptimal integration in MD participants.

## Discussion

This study investigated whether combining binocular disparity and motion parallax can improve depth perception in participants with central field loss due to macular degeneration and how individual visual characteristics modulate this ability. Our findings reveal that cue integration benefits depend on scotoma characteristics, with important implications for understanding depth processing adaptations in central field loss.

In normally sighted participants, we replicated previous findings that disparity induces more precise depth judgments than motion parallax alone (Bradshaw & Rogers, 1996; Hartle & Wilcox, 2021; McKee & Taylor, 2010; Rogers & Graham, 1982). Furthermore, we also observed that combining disparity with motion parallax significantly improved depth discrimination, even in the presence of simulated central scotomas up to at least 6° of diameter. These results confirm that depth cue integration mechanisms are very robust in individuals without visual impairments (Bradshaw & Hibbard, 1998; Bradshaw & Rogers, 1996).

Our methodological approach, based on Scheller and Nardini's (2024) work, provided a robust framework for assessing cue integration by determining the better and worse individual cues for each individual and comparing these to the maximum likelihood estimation predictions. For control participants, thresholds for disparity alone were already low, and MLE predicted a limited benefit from combining both cues, especially in the absence of an artificial scotoma. Yet, control participants demonstrated optimal or “super-optimal” integration (i.e., better than predicted) when disparity was added to motion parallax set at threshold (disparity + Th_parallax_), which suggests that the two cues may interact nonlinearly. Conversely, control participants showed limited integration when motion parallax was added to disparity at threshold (parallax + Th_disparity_), which was expected from our model. In this case, the combined threshold was not significantly different from the better cue. Moreover, for most control participants, the MLE model better predicted the thresholds for the combined conditions compared to the winner-take-all model, indicating a real integration of both cues. Some exceptions have to be noted: When the sensitivity to one cue (usually disparity) was much higher than the sensitivity to the other cue, the winner-take-all was occasionally a better fit. This asymmetric integration pattern, where integration depends on which cue serves as the primary signal, has important implications for understanding cue combination mechanisms and interpreting results from the MD group.

MD participants exhibited markedly different patterns of results compared to controls. Thresholds were higher than controls overall, consistent with previous research showing impaired stereopsis in central field loss (Verghese et al., 2022; Verghese & Ghahghaei, 2020), and the large variability between MD participants highlighted the clinical heterogeneity of this population. This variability was far greater than that observed in controls and necessitated individual-level analyses.

Cue integration analyses revealed that integration success was strongly determined by scotoma characteristics, and we identified distinct patterns across clinical subgroups. MD participants with large stereo and binocular scotomas had the most variability among the subgroups, but interestingly, two of them (MD1 and MD2, out of four patients) showed a better than predicted combined threshold when disparity was used as a primary cue, despite their extensive central field loss, suggesting that peripheral coarse stereopsis can support effective cue combination even with large central scotomas and even if the thresholds are higher than those observed in controls. For these two patients, the cue integration model better predicted their combined threshold in the disparity + Th_parallax_, while the winner-take-all model better predicted the performance of the two other patients. Moreover, two patients (MD1 and MD4) had better thresholds in the combined condition parallax + Th_disparity_ compared to motion parallax alone, but the benefit from the combination of both cues was not considered optimal using the MLE model. However, it is important to note that for this condition (parallax + Th_disparity_), the winner-take-all model better explains every participant's thresholds. Overall, these results suggest an asymmetric integration, depending on which cue serves as a primary signal.

MD participants with small binocular scotoma and small stereo scotoma demonstrated integration patterns most similar to controls. In most cases, combining disparity with motion parallax at the threshold led to a lower threshold in the combined condition, and this benefit was often better than predicted by the MLE model. Importantly, for all of these MD participants, the cue integration model predicted the combined thresholds better than the winner-take-all model, which indicates a real integration of both cues. This finding suggests that when sufficient visual function remains, the fundamental mechanisms of cue integration are preserved. Conversely, when motion parallax served as a primary cue, most MD participants also benefited from combining both cues, but the winner-take-all model explained their thresholds better compared to the MLE predictions.

On the other hand, the MD participant with compromised binocular function due to amblyopia (strabismus) showed the most impaired integration, as expected, with suboptimal performance and similar thresholds when cues were combined or presented alone. This confirms that effective cue integration requires functional binocular vision, even if that vision is somewhat degraded.

A critical finding was the differential impact of central field loss on the two depth cues. Motion parallax thresholds showed no significant correlations with any clinical measures (see Figure 6), including scotoma size, PRL eccentricity, or stereoacuity, highlighting that this monocular cue remains relatively preserved in macular degeneration. This preservation is consistent with previous findings (Guénot et al., 2022; Shanidze & Verghese, 2019) and reflects motion parallax's reliance primarily on motion perception, which appears less sensitive to eccentricity than retinal image disparity.

In contrast, all conditions involving disparity (whether used alone or combined with motion parallax) showed strong correlations with stereo scotoma size (see Figure 6). This is consistent with the known degradation of disparity processing with eccentricity (8–10 times higher thresholds at 10° compared to fovea; Blakemore, 1970; Fendick & Westheimer, 1983). This differential preservation reinforces the potential of motion parallax as a compensatory cue in MD participants with extensive central field loss.

Furthermore, the asymmetric integration patterns observed in both controls and MD participants, where integration occurred predominantly when motion parallax (at threshold) was added to disparity rather than vice versa, may reflect fundamental principles of how the visual system combines depth cues of different reliabilities. In MD participants, this asymmetry was more pronounced, with suboptimal integration observed in 4 of the 10 MD participants in the parallax + Th_disparity_ condition. This suggests that individuals with MD may overrely on disparity when available, despite its degradation, rather than flexibly reweighting cues based on their actual reliability. An alternative explanation could partially emerge from our experimental design, in which one cue was fixed at its 75% threshold while the other was varied. This simplified approach was intentionally chosen to reduce testing time and participant fatigue, given that our study involved older adults and patients with macular degeneration. Given that the parallax threshold (when converted into equivalent depth units) was about 10 times the disparity threshold for controls, one might speculate that the two cues at threshold provided asymmetric depth information and that the addition of the parallax threshold to disparity was a better depth cue than the addition of threshold disparity to motion parallax. Nevertheless, if the asymmetric pattern came only from our design, we could expect that adding threshold disparity to motion parallax would have little to no effect on the combined condition parallax + Th_disparity_, but this was not the case in general. For instance, in control participants, the combined threshold parallax + Th_disparity_ was significantly lower than the threshold for motion parallax alone for every scotoma size and even when the integration was not considered optimal.

Another important factor to consider, especially when using motion parallax as a depth cue, is smooth pursuit. MD participants’ analyses provided additional information about motion parallax processing difficulties. Consistent with previous studies (Shanidze et al., 2016; Shanidze et al., 2022), MD participants showed more saccades compared to controls, as well as an increased onset latency. The average pursuit gain for MD participants was comparable to that of controls, but some variability was observed, with a minimum of 0.63 for MD5 to a maximum of 1.61 for MD6. Since accurate smooth pursuit is crucial for extracting depth from motion parallax (Nawrot, 2003; Nawrot & Joyce, 2006), these pursuit deficits may contribute to the suboptimal integration observed when motion parallax serves as the primary cue. However, no significant correlations between pursuit metrics and thresholds were found, which suggests that other factors also contribute to integration difficulties.

Altogether, these findings have important implications for understanding depth processing adaptations in central field loss. It indicates that participants with central field loss benefit from combining motion parallax and disparity, even in the presence of a large stereo scotoma. This ability is contingent upon residual coarse stereopsis. Furthermore, the presence of a large stereo scotoma may limit access to disparity cues, and impaired pursuit may limit effective use of depth information from motion parallax. For this reason, future work should explore rehabilitation strategies to enhance depth perception in MD, potentially by training individuals with MD to better utilize motion parallax and improve smooth pursuit. It is also important to note that in real-world scenarios, disparity and motion parallax are not independent cues, as both arise from the same three-dimensional structure of the scene and may share correlated noise, potentially limiting the benefit that could arise from their combination. Real-world tasks involving navigation and object manipulation could also help assess the functional relevance of these findings. Additionally, the strong predictive value of stereo scotoma size for disparity-based performance suggests that this clinical measure could be useful for predicting depth perception outcomes and personalizing rehabilitation strategies in MD to individual visual profiles.

Finally, it is important to note that our study focused on simple depth discrimination tasks. Therefore, it is still unclear how these results translate to everyday activities such as navigation or object manipulation. Additionally, the heterogeneity of the macular degeneration population, while revealing important individual differences, also limited our ability to detect subtle group-level effects. Future studies with larger, more homogeneous clinical groups would help confirm these findings and explore other factors that influence how successfully participants with MD integrate visual cues for depth.

## Conclusions

Participants with central field loss due to macular degeneration show variable but significant capacity for depth cue integration, with outcomes strongly determined by scotoma characteristics and residual binocular function. Motion parallax emerges as a potentially valuable compensatory cue, while the ability to combine depth cues depends critically on residual peripheral stereopsis. These findings highlight the importance of individual approaches to vision rehabilitation and suggest that targeted programs focused on depth from motion parallax could be beneficial for individuals with MD who have extensive stereo scotoma.
